# Association of cause-specific hospital admissions with high and low temperatures in Thailand: a nationwide time series study

**DOI:** 10.1016/j.lanwpc.2024.101058

**Published:** 2024-04-04

**Authors:** Bo Wen, Wissanupong Kliengchuay, San Suwanmanee, Htoo Wai Aung, Narut Sahanavin, Weerayut Siriratruengsuk, Sawaeng Kawichai, Benjawan Tawatsupa, Rongbin Xu, Shanshan Li, Yuming Guo, Kraichat Tantrakarnapa

**Affiliations:** a Climate, Air Quality Research Unit, School of Public Health and Preventive Medicine, Monash University, Melbourne, VIC, Australia; b Department of Social and Environmental Medicine, Faculty of Tropical Medicine, Mahidol University, Bangkok, Thailand; c Environment, Health and Social Impact Unit, Faculty of Tropical Medicine, Mahidol University, Bangkok, Thailand; d Department of Epidemiology, Faculty of Public Health, Mahidol University, Bangkok, Thailand; e Faculty of Physical Education, Srinakharnwirot University, Nakhon Nayok, Thailand; f School of Health Science, Mae Fah Lhuang University, Chiang Rai, Thailand; g Research Institute of Health Science, Chiang Mai University, Chiang Rai, Thailand; h Department of Health, Ministry of Public Health, Nonthaburi, Thailand

**Keywords:** High and low temperatures, Cause-specific hospital admissions, Nationwide study

## Abstract

**Background:**

Non-optimum temperatures are associated with a considerable mortality burden. However, evidence of temperature with all-cause and cause-specific hospital admissions in tropical countries like Thailand is still limited.

**Methods:**

Daily all-cause and cause-specific hospital admissions for outpatient and inpatient visits were collected from 77 provinces in Thailand from January 2013 to August 2019. A two-stage time-series approach was applied to assess the association between non-optimum temperatures and hospital admission. We first fitted the province-specific temperature-morbidity association and then obtained the national association in the second stage using a random-effects meta-analysis regression. The attributable fraction (AF) of hospital admissions with 95% empirical confidence interval (eCI) was calculated.

**Findings:**

A total of 878,513,460 all-cause outpatient admissions and 32,616,600 all-cause inpatient admissions were included in this study. We observed a J-shaped relationship with the risk of hospital admissions increasing for both cold and hot temperatures. The overall AFs of all-cause hospital admissions due to non-optimum temperatures were 7.57% (95% eCI: 6.47%, 8.39%) for outpatient visits and 6.17% (95% eCI: 4.88%, 7.20%) for inpatient visits. Hot temperatures were responsible for most of the AFs of hospital admissions, with 6.71% (95% eCI: 5.80%, 7.41%) for outpatient visits and 4.50% (95% eCI: 3.62%, 5.19%) for inpatient visits. The burden of hospital admissions was greater in females and in children and adolescents (0–19 years). The fractions of hospital admissions attributable to non-optimum temperatures exhibited variation among disease categories and geographical areas.

**Interpretation:**

The results indicate that low and high temperature has a significant impact on hospital admissions, especially among the females, and children and adolescents (0–19 years). The current investigation could provide evidence for policymakers to develop adaptation strategies and mitigate the adverse effects of climate change on public health in Thailand and other tropical countries.

**Funding:**

10.13039/501100004704National Research Council of Thailand (NRCT): E-Asia Joint Research Program: Climate change impact on natural and human systems (N33A650979).

**Research in context**

**Evidence before this study**

Non-optimal temperatures are a recognized significant contributor to global health burden, affecting various diseases. We searched PubMed, Scopus, Web of Science, and Google Scholar for studies published in English between database inception and October 20, 2023, that explore the temperature-related mortality burden. We used a combination of search terms, including “temperature”, “morbidity, “hospitalization”, “hospital admission”, “inpatient” and “outpatient”. The search identified highly assessed health outcomes in previous studies, including cardiovascular diseases, respiratory diseases, and mental health, with other causes of hospital admission largely unexplored. Additionally, most previous research mainly quantified temperature-related hospitalizations within specific countries (e.g., Australia, Spain, Brazil, and China). Currently, no nationwide study in Thailand has examined the association between the temperature and hospital admission.

**Added value of this study**

This study fills a crucial gap by providing the first national overview of cause-specific hospitalizations attributable to non-optimal temperatures in Thailand. Our findings show that non-optimum temperatures contribute substantially to hospital admissions, primarily driven by heat exposure. However, cold temperatures contribute to a larger proportion of inpatient admissions from mental and behavioral, nervous, cardiovascular, maternal, and perinatal diseases. Furthermore, we observed a higher burden of hospital admissions among females and children/adolescents. These comprehensive findings on both all-cause and cause-specific hospital admissions offer valuable insights to mitigate healthcare utilization impacts and inform future policy development.

**Implications of all the available evidence**

In the context of climate change, more frequent extreme temperatures necessitate the development of more robust strategies and policies to prevent temperature-related hospitalizations. This study contributes to understanding the impact of temperature events on vulnerable populations in subtropical and tropical climates like Thailand. The findings highlight the need for targeted interventions across various causes of hospital admissions. For example, cold-related adaptations warrant greater emphasis on cardiovascular, maternal, and perinatal diseases. Additionally, interventions should prioritize high-susceptibility groups, including females and children/adolescents.

## Introduction

The World Health Organization (WHO) reported that the global mean temperature in 2021 was warmer than previous decades, with an increase of 1.11 ± 0.13 °C compared to pre-industrial periods.^1^ These temperature variations have been linked to detrimental effects on human health, affecting both mortality and morbidity rates. Between the years 2000 and 2019, non-optimum ambient temperatures led to more than 5 million fatalities worldwide. Heat exposure, in particular, contributed significantly to approximately 0.91% of the overall mortality rate.^2^^,^^3^

Emerging evidence has documented a U-shaped or V-shaped pattern of the association between temperatures and health outcomes, implying that both excessively high and low temperatures significantly increase disease risks. For example, Bobb et al. linked extreme heat to increased admissions for various conditions, including heat stroke, fluid and electrolyte imbalance, and renal failure.^4^ Similarly, cold exposure has also been identified as a critical risk factor for cardiovascular and respiratory diseases.^5^^,^^6^ Notably, while previous studies have predominantly focused on outcomes like cardiovascular and respiratory diseases, other causes of temperature-related hospital admissions remain largely understudied.

Thailand presents a specific case study. Its annual mean temperature of 26.3 °C exhibits seasonal variations of 5.7 °C, with a concerning trend of rising warm nights. Projections indicate that Thailand's temperature is expected to increase by approximately 0.95 °C–3.23 °C by the year 2090, compared to the baseline of 1986–2005.^7^ A study has shown that every 1 °C increase above the 29 °C threshold in Chiang Mai was associated with 26.3% higher visits for diabetes and 19.2% higher visits for circulatory diseases.^8^ Despite these initial findings, no nationwide study in Thailand has comprehensively examined the association between temperature and hospital admissions. More importantly, evidence for the relationship between temperature and hospital admission in tropical regions is still limited.

Recognizing the need for a deeper understanding of this critical issue, our study aims to investigate the association between both high and low temperatures and daily hospital admissions across Thailand from 2013 to 2019. By filling this vital research gap, we hope to elucidate the comprehensive impact of temperature on hospital admissions in Thailand, considering its unique climate and health equity challenges. This knowledge can empower policymakers to promote health equity and develop targeted adaptation and mitigation measures to protect public health in the face of rising temperatures, especially in the tropical setting.

## Methods

### Data

#### Health data

Thailand is a tropical country located in Southeast Asia. Daily data on hospital admissions of inpatient and outpatient visits for illnesses from January 2013 to August 2019 were collected from the computerized database of the Ministry of Public Health in Thailand, which were validated by the health information management system of Thailand. Briefly, this database included all admissions from primary, secondary, and tertiary hospitals from the Universal Coverage Scheme (UCS) across 77 provinces in Thailand, covering over 76% of Thai population.^9^^,^^10^ The National Health Security Office (NHSO) further reviewed and improved the quality of the data.^9^ We further cleaned the data, including removing cases with missing or unreasonable values for gender and age. The specific reasons for hospital admissions inpatient and outpatient visits were classified using the tenth revision of the International Classification of Diseases (ICD-10), which encompassed various diseases such as Infectious and parasitic (A00-B99), Neoplasms (C00-D49), Endocrine, nutritional, and metabolic (E00-E90), Mental and behavioral (F01-F99), Nervous (G00-G99), Cardiovascular (I00-I99), Respiratory (J00-J99), Digestive (K00-K93), Skin (L00-L99), Musculoskeletal (M00-M99), Genitourinary (N00-N99), Maternal (O00-O99), and Perinatal (P00-P96).

#### Meteorological data

Hourly temperature and dew point temperature data were obtained from the European Centre for Medium-Range Weather Forecasts Reanalysis v5 (ERA-5) reanalysis dataset with a spatial resolution of 0.1° × 0.1°.^11^ Relative humidity (RH) was computed using mean ambient temperature and dew point temperature.^12^ We calculated the daily mean temperature and daily mean RH for each grid by averaging the hourly data by calendar days. The population-weighted temperature and RH were then calculated for each province by averaging the values of all grid cells overlaying the province weighted by the population distribution (https://www.worldpop.org).

### Statistical analysis

#### Descriptive analyses

The distributions of daily number of all-cause and cause-specific hospital admissions for inpatient and outpatient and meteorological variables across 77 provinces were reported by 25th percentile, mean, standard deviation (SD), and 75th percentile. We then calculated the daily mean temperature for each province during the study period and presented the map to show the geographical distribution of ambient temperature in Thailand.

#### Associations between hospital admissions and temperature

A two-stage meta-analytical strategy was used to estimate the risk of hospital admissions associated with high and low temperatures. In the first stage, we constructed a generalized linear regression model with a quasi-Poisson family for each province to obtain province-specific estimates, using the logarithm of annual province population as an offset. The distributed lag non-linear model (DLNM) was constructed with a cross-basis function applied to temperature.^13^, ^14^, ^15^

$$Y_{it}∼poisson\left(\mu_{it}\right)\log\left(\mu_{it}\right)=\alpha +cb\left(Temp_{it},lag=21\right)+ns\left(RH_{it},df=3\right)+ns\left(Time_{it},df=8\;per\;year\right)+\beta DOW_{it}$$

where $Y_{it}$ is the number of hospital admissions on day t in province i. a is the intercept. cb is the cross-basis function of temperature, featuring both the exposure-response association and the lag-response association. The parameters in the model were selected based on previous studies.^13^^,^^16^, ^17^, ^18^ For exposure-response association, a cubic B-spline with three internal knots placed at the 10th, 75th, and 90th percentiles of province-specific temperature distributions was applied to capture potential nonlinearities and directional changes in the temperature-hospitalization relationship, particularly at temperature extremes.^13^^,^^16^ For the lag-response association, a natural cubic spline with an intercept and three internal knots placed at equally spaced values in the log scale was used to model the lag-response association up to 21 days. This knot placement choice allowed more flexibility in the first lag period.^13^^,^^17^^,^^18^ A maximum lag of 21 days was widely used in previous studies to account for the potentially delayed effects of heat and cold exposure while minimizing the influence of “harvesting” effects.^3^^,^^13^^,^^18^ RH was controlled in the model using a natural cubic spline with three degrees of freedom (df). In addition, the long-term trend and seasonality were controlled using a natural cubic spline of Time with eight degrees of freedom (df) per year in the models. We also controlled the day of the week (DOW). The analyses were repeated in different subgroups, including sex (females and males) and age groups (0–19, 20–39, 40–59, 60–79, 80+ years).

In the second stage, we applied a random-effects meta-analysis model to pool the province-specific estimations and obtain the national associations between non-optimum temperatures and hospital admissions. Predictors at the province level were included in the meta-regression, including the average temperature, the range of temperature, and the annual Gross domestic product (GDP) per capita. The best linear unbiased predictions (BLUPs) based on the meta-regression model were used to re-estimate the province-specific associations, which would borrow information across provinces within the same hierarchical level in the meta-regression, potentially mitigating the issue of limited data in some provinces and ultimately leading to more accurate and precise estimates.^18^^,^^19^ The weight assigned to each province in the analysis is inversely proportional to a combined measure of uncertainty from two sources: (1) within-study standard error (a function of both sample size and within-study exposure variation), and (2) between-study heterogeneity, reflecting potential differences in effect estimates across provinces.^20^

#### Attributable fractions

We defined the minimum risk temperature (MRT) as the temperature with the lowest risk of hospital admissions. The association between temperature and hospital admissions was then re-centered using the MRT as the reference and the relative risk (RR) at any given temperature was calculated for each province. To capture the burden of hospital admissions, the temperature on each day and the MRT were used to calculate the attributable fraction (AF) and attributable number (AN) of hospital admissions for that day and the corresponding lag periods (21 days).^18^^,^^21^ A forward method was used with the following equations:

$$AF_{it}=1-\exp\left(-\sum_{l=0}^{21}\beta_{Temp_{t},l}\right)$$

$$AN_{it}=AF_{it}\times \sum_{l=0}^{21}\frac{n_{t+l}}{21+1}$$

where $\sum_{l=0}^{21}\beta_{Temp_{t},l}$ stands for the overall cumulative log-relative risk for temperature in day *t* ($Temp_{t}$) in province *i*, *l* is the lag day considered with a maximum value of 21.

The total number of hospital admissions attributed to non-optimum temperatures was then obtained by summing the AN of all the days. We then calculated the total AF by dividing the total AN by the total number of hospital admissions. The empirical confidence intervals (eCIs) for attributable burden were estimated using the Monte Carlo simulations. Four types of non-optimum temperatures were defined according to the MRT and percentiles of the province-specific temperature distribution: extreme cold (<2.5th percentile), moderate cold (from 2.5th percentile to the MRT), moderate hot (from MRT to 97.5th percentile), and extreme hot (>97.5th percentile).

#### Sensitivity analyses

Sensitivity analyses were performed to test the robustness of our results. First, the maximum number of days for the lag period was changed from 21 days to alternative selections (19–23 days). Second, the df for the long-term trend and seasonality was replaced by alternative selections (6–10 df/year). Third, the diurnal temperature range (DTR) was further adjusted in the model to account for the effect of temperature variation. Finally, we used the penalized spline function for RH in the model instead of the natural spline function.

Ethical approval was not formally required for this study, as the analysis was conducted on aggregate data at a group level without contacting individual participants. We performed all the analyses using the R software (version 4.0.3), with the “dlnm” and “mixmeta” packages used for the construction of the cross-basis function and the meta-analyses.

### Role of the funding source

The funders of the study had no role in study design, data collection, data analysis, data interpretation, or writing of the report. The corresponding author had full access to all the data in the study and had final responsibility for the decision to submit it for publication.

## Results

### Descriptive statistics

**Fig. 1:**
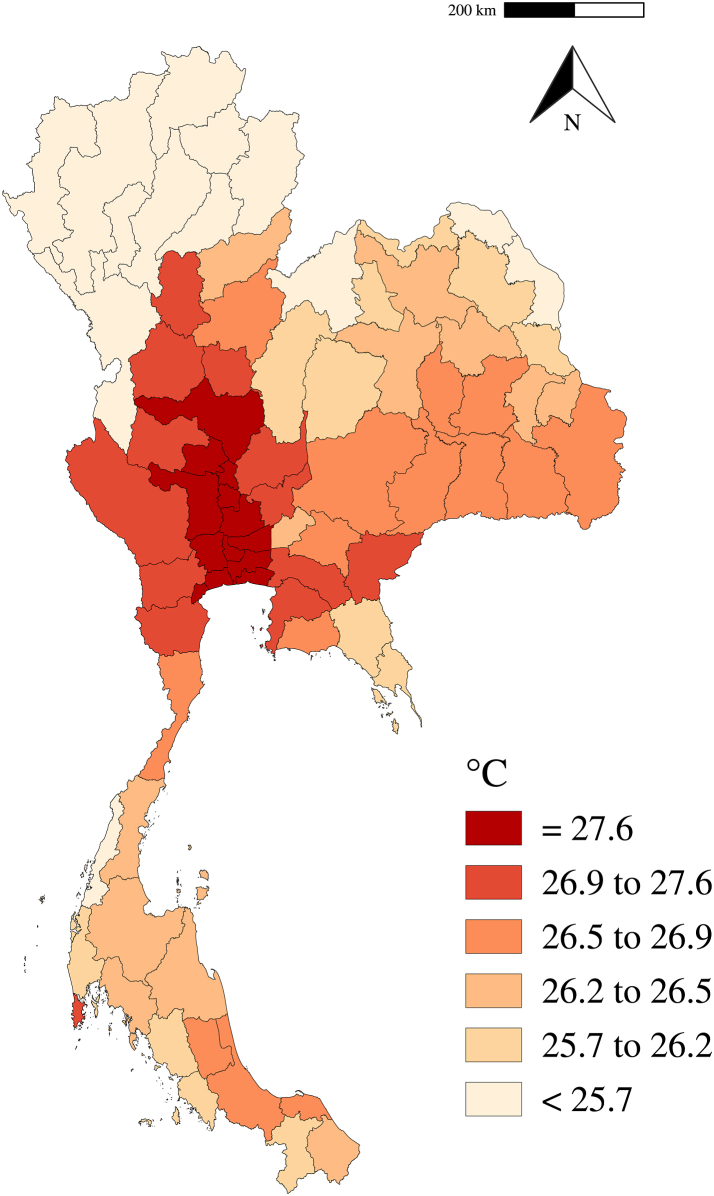
**Annual mean temperature of 77 provinces in Thailand during 2013–2019**.

The descriptive statistics on temperature and hospital admissions are presented in Table 1. A total of 878,513,460 all-cause outpatient admissions and 32,616,600 all-cause inpatient admissions were included in this study, of which 128,238,863 and 2,659,264 for cardiovascular diseases, and 139,927,844 and 5,025,365 for respiratory diseases, respectively. The daily average of outpatient admissions was 5114 (standard deviation [SD]: 4369), while the daily average of inpatient admissions was 193 (SD: 152). We recorded an annual mean temperature of 26.5 °C and an annual mean RH of 73.6%. The spatial distribution of annual mean temperatures is presented in Fig. 1. The annual mean temperature across different provinces represents a distinct tropical climate, ranging from 22.7 °C in Mae Hong Son to 28.2 °C in Ang Thong.

**Table 1:** Descriptive statistics on the average number of daily hospital admission and weather across 77 provinces in Thailand.

	Outpatient				Inpatient			
	Total	25th percentile	Mean (SD)	75th percentile	Total	25th percentile	Mean (SD)	75th percentile
All-cause	878,513,460	1816	5114 (4369)	6831	32,616,600	91	193 (152)	252
Infectious and parasitic	40,200,847	90	234 (206)	310	4,202,827	11	25 (21)	33
Endocrine, nutritional and metabolic	99,225,758	74	579 (618)	868	1,133,756	3	7 (6)	10
Cardiovascular	128,238,863	111	748 (748)	1143	2,659,264	7	16 (13)	21
Respiratory	139,927,844	352	815 (662)	1065	5,025,365	14	30 (24)	39
Digestive	112,424,459	231	655 (566)	906	3,148,494	8	19 (15)	25
Skin	31,659,355	78	185 (155)	244	878,097	2	6 (5)	8
Musculoskeletal	112,743,578	209	657 (604)	890	870,461	2	6 (6)	8
Genitourinary	37,744,665	66	220 (215)	295	2,495,622	6	15 (13)	20
Maternal	4,740,907	11	28 (26)	36	3,865,731	11	23 (17)	32
Perinatal	1,220,392	2	8 (10)	10	1,158,627	3	8 (6)	10
Neoplasms	11,518,016	9	69 (122)	78	1,737,902	3	11 (15)	13
Mental and behavioural	25,925,756	22	152 (198)	204	612,694	1	5 (6)	5
Nervous	14,971,305	27	87 (86)	119	582,187	2	4 (3)	5
Temperature (°C)		25.3	26.5 (2.5)	27.9				
Relative humidity (%)		64.8	73.6 (12.6)	83.6				

Note: The distributions of daily number of all-cause and cause-specific hospital admissions for inpatient and outpatient and meteorological variables across 77 provinces were reported by 25th percentile, mean, standard deviation (SD), and 75th percentile.

### Associations between hospital admissions and temperature

**Fig. 2:**
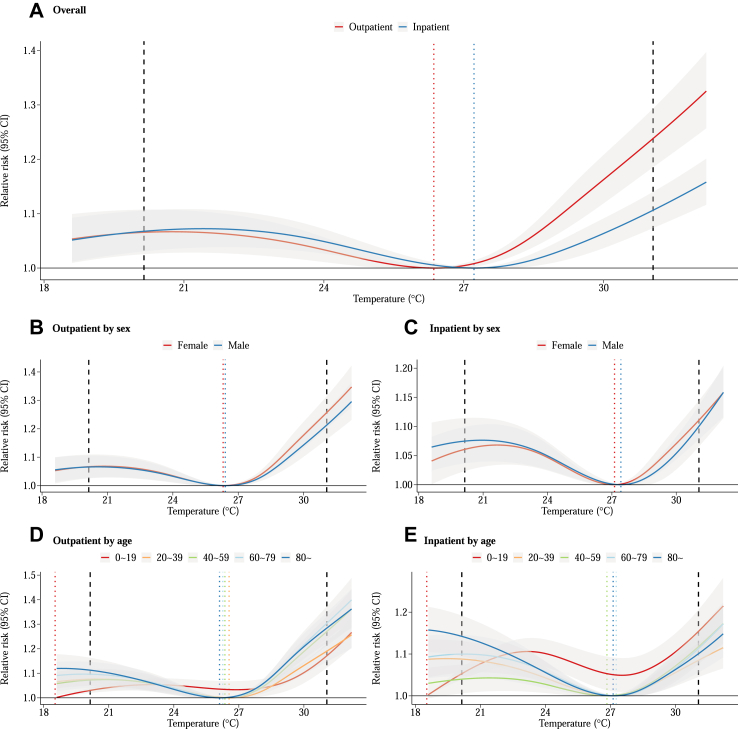
**The exposure-response association between temperature and hospital admission in Thailand.** A: Overall; B and C: stratified results by sex; D and E: stratified results by age groups. Grey error areas represent 95% CIs. The black dashed lines are placed at the 2.5th and 97.5th percentiles of the temperature distribution. The vertical lines with different colors (dotted) are placed at the minimum risk temperature (MRT).

Fig. 2 illustrates the cumulative exposure-response curves for the associations between temperature and outpatient as well as inpatient hospital admissions. We observed a J-shaped relationship with the risk of hospital admissions increasing for both cold and hot temperatures (Fig. 2A). The relationship between temperature and hospital admissions exhibited uniformity among various demographic groups (Fig. 2B–E), such as sex and age, with the notable exception of the age group 0–19 years. The exposure-response curve for those aged 0–19 years showed an overall upward trend, despite some fluctuations. The minimum risk temperature (MRT) was 26.4 °C and 27.2 °C for outpatient and inpatient hospital admissions, respectively. Compared to the MRT, the overall relative risk (RR) of all-cause hospital admissions at the extreme hot temperature (97.5th of the temperature distribution) was 1.24 (95% confidence interval [CI]: 1.19, 1.29) for outpatient and 1.11 (95% CI: 1.07, 1.14) for inpatient. By contrast, the overall RR at the extreme cold temperature (2.5th of the temperature distribution) was 1.07 (95% CI: 1.03, 1.11) for both outpatient and inpatient hospital admissions, which was lower than that at the extreme hot temperature. The exact values for MRT and RRs are presented in supplementary Table S1. The exposure-response curves for each cause were presented in supplementary Figs. S1 and S2.

### Attributable burden

Table 2 presents the fractions of hospital admissions attributable to non-optimum temperatures in Thailand. The overall attributable fractions of all-cause hospital admissions were 7.57% (95% empirical confidence interval [eCI]: 6.47%, 8.39%) for outpatient visits and 6.17% (95% eCI: 4.88%, 7.20%) for inpatient visits. Hot temperatures were responsible for most of the attributable fractions of hospital admissions, with 6.71% (95% eCI: 5.80%, 7.41%) for outpatient visits and 4.50% (95% eCI: 3.62%, 5.19%) for inpatient visits. The overall fractions for females (8.28% for outpatient and 7.25% for inpatient) were higher than the fractions for males (6.63% for outpatient and 5.49% for inpatient). Compared with other age groups, children and adolescents (0–19 years) have the highest attributable fractions, with 10.92% for outpatient and 16.43% for inpatient.

**Table 2:** Attributable fraction (%) of hospital admission associated with low and high temperatures in Thailand.

	Total	All heat	Extreme heat^a^	All cold	Extreme cold^b^
Outpatient	7.57 (6.47, 8.39)	6.71 (5.80, 7.41)	1.44 (1.36, 1.49)	0.86 (0.67, 0.98)	0.21 (0.14, 0.24)
Male	6.63 (5.53, 7.41)	5.72 (4.80, 6.36)	1.33 (1.25, 1.39)	0.92 (0.73, 1.04)	0.22 (0.15, 0.24)
Female	8.28 (7.22, 9.10)	7.45 (6.57, 8.15)	1.51 (1.44, 1.57)	0.82 (0.65, 0.94)	0.20 (0.13, 0.23)
0 ∼ 19 years	10.92 (9.94, 11.73)	10.01 (9.26, 10.63)	1.10 (1.06, 1.14)	0.91 (0.68, 1.10)	0.09 (0.05, 0.11)
20 ∼ 39 years	6.36 (5.44, 7.13)	5.37 (4.62, 6.02)	1.25 (1.18, 1.30)	0.98 (0.82, 1.10)	0.21 (0.15, 0.24)
40 ∼ 59 years	8.30 (7.12, 9.14)	7.48 (6.50, 8.19)	1.59 (1.51, 1.64)	0.81 (0.63, 0.95)	0.20 (0.12, 0.23)
60 ∼ 79 years	8.04 (6.91, 8.81)	6.63 (5.75, 7.24)	1.66 (1.55, 1.72)	1.41 (1.16, 1.57)	0.35 (0.24, 0.38)
80 ∼ years	8.20 (6.90, 9.14)	6.53 (5.51, 7.34)	1.61 (1.49, 1.67)	1.67 (1.39, 1.80)	0.45 (0.32, 0.48)
Inpatient	6.17 (4.88, 7.20)	4.50 (3.62, 5.19)	0.96 (0.86, 1.02)	1.67 (1.26, 2.00)	0.24 (0.16, 0.28)
Male	5.49 (4.28, 6.39)	3.04 (2.33, 3.57)	0.95 (0.83, 1.02)	2.45 (1.95, 2.81)	0.35 (0.26, 0.41)
Female	7.25 (6.00, 8.22)	5.90 (4.99, 6.60)	1.01 (0.91, 1.07)	1.35 (1.01, 1.62)	0.19 (0.12, 0.24)
0 ∼ 19 years	16.43 (14.50, 17.78)	15.52 (14.25, 16.44)	1.20 (1.09, 1.25)	0.91 (0.25, 1.33)	0.10 (0.05, 0.14)
20 ∼ 39 years	6.37 (4.29, 7.75)	3.37 (2.26, 4.08)	0.79 (0.63, 0.88)	3.00 (2.03, 3.67)	0.43 (0.31, 0.49)
40 ∼ 59 years	4.89 (3.56, 5.89)	3.80 (3.00, 4.40)	1.00 (0.87, 1.08)	1.09 (0.56, 1.49)	0.18 (0.10, 0.24)
60 ∼ 79 years	5.36 (4.34, 6.13)	2.52 (1.97, 2.92)	1.03 (0.93, 1.09)	2.85 (2.37, 3.22)	0.47 (0.35, 0.54)
80 ∼ years	5.90 (4.50, 7.05)	2.50 (1.89, 3.03)	1.01 (0.85, 1.10)	3.40 (2.61, 4.03)	0.67 (0.53, 0.73)

a Extreme heat is defined as temperature greater than 97.5th percentile of the province-specific temperature distribution.

b Extreme cold is defined as temperature lower than 2.5th percentile of the province-specific temperature distribution.

**Fig. 3:**
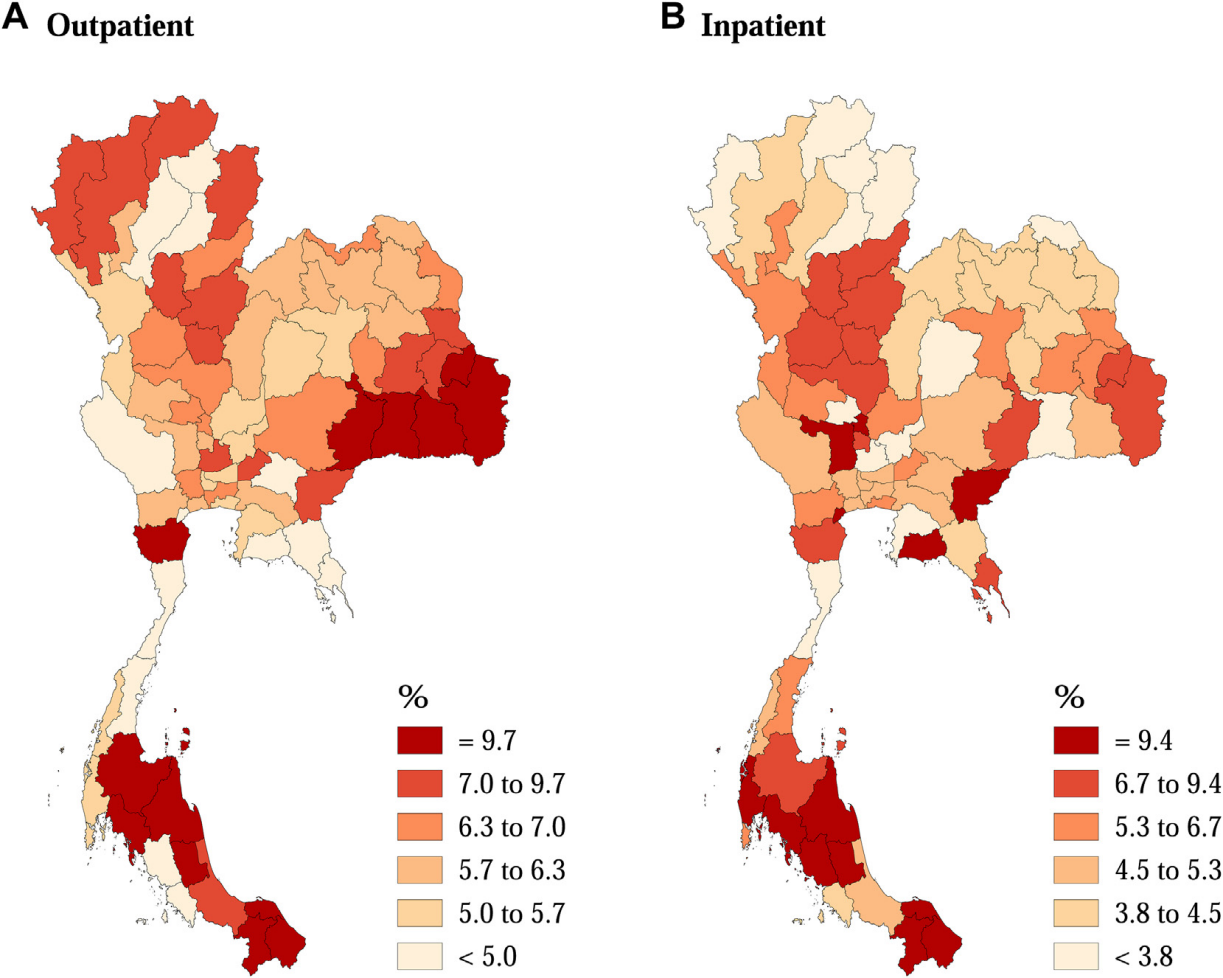
**Attributable fraction (%) of outpatient (A) and inpatient (B) admissions associated with low and high temperatures in 77 provinces of Thailand**.

**Fig. 4:**
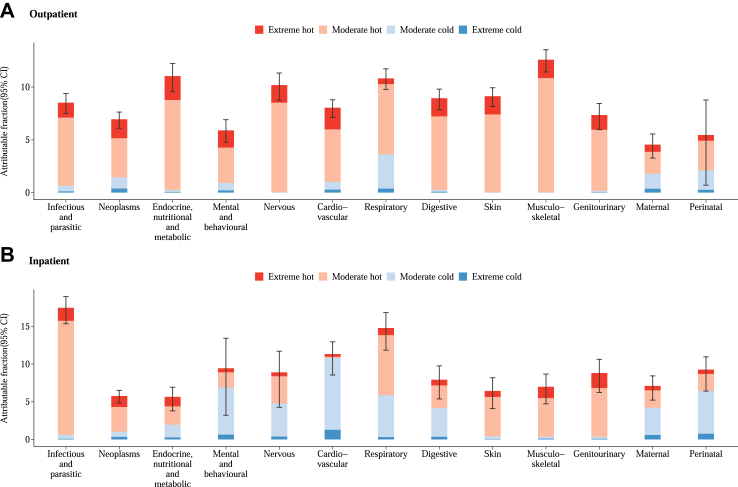
**Attributable fraction (%) of cause-specific outpatient (A) and inpatient (B) admissions associated with low and high temperatures.** Bar charts present the point estimates with error bars for 95% CIs.

The fractions of all-cause hospital admissions attributable to non-optimum temperatures varied geographically, with the highest fractions for both outpatient and inpatient visits observed in the Southern Thailand (Fig. 3). Meanwhile, the lowest estimates of attributable fractions were reported in Eastern Thailand for outpatient visits and in Northern Thailand, Central Thailand, and Bangkok and Vicinities for inpatient visits. Fig. 4 illustrates the fractions of cause-specific hospital admissions attributable to different components of non-optimum temperatures. The highest fractions of outpatient hospital admissions were observed for musculoskeletal (12.6%) and endocrine, nutritional and metabolic diseases (11.0%), while the fractions of inpatient were highest for infectious and parasitic (17.5%) and respiratory diseases (14.8%). Heat-related hospital admissions contributed to the most attributable fractions for almost all causes, except for cardiovascular, mental and behavioral, maternal, and perinatal diseases for inpatient visits. Our findings remain robust in the sensitivity analyses after changing the maximum number of days for the lag period (supplementary Table S2), the df for the long-term trend and seasonality (supplementary Table S3), controlling for DTR (supplementary Table S4), and using penalizaed spline function for RH (supplementary Table S5).

## Discussion

To the best of our knowledge, this is the first nationwide study to evaluate the impacts of non-optimum temperatures on hospital admissions for both outpatient and inpatient visits in Thailand. Our findings suggested that non-optimum temperatures caused a substantial fraction of hospital admissions, with heat being the primary contributor. The results identified that the burden of hospital admissions was greater in females and in children and adolescents. In addition, our results indicated that the burden of hospital admissions exhibited variation among disease categories and geographical areas.

Consistent with previous studies, we observed a non-linear relationship between temperature and hospital admissions, wherein the risk of hospital admissions increased with temperatures below the MRT and above the MRT.^13^^,^^22^^,^^23^ However, our analyses indicated that heat exposure was associated with greater risk and burden of hospital admissions in Thailand, whereas the cold effects tended to be more profound in previous studies. For example, a global study by Zhao et al. reported that 9.43% of all deaths were associated with all death, with 8.52% attributed to cold and 0.91% attributed to heat.^3^ By contrast, a previous investigation conducted in Chiang Mai of Thailand also observed a higher risk of outpatient visits at high temperatures, which was consistent with our findings.^8^ Another study reported that extreme high temperatures have greater risks of all-cause mortality by assessing three tropical cities in the Philippines.^24^ The findings indicated that health is more adversely affected by heat exposure in sub-tropical climate. Thailand will experience a warmer weather in the context of climate change, leading to an anticipated escalation in the health burden.^25^ Adaptation interventions should be accelerated to mitigate the heat-related challenges, especially in the health sector, such as providing climate services to the health sector and conducting vulnerability assessments.^26^^,^^27^

In this study, we found that the impacts of non-optimum temperatures were more profound in females, resulting in higher fractions of hospital admissions. Our analysis revealed that the heat-related attributable fractions were substantially higher among females compared to males, indicating females may be more vulnerable to heat exposure. This could be related to the physiological differences in coping with heat stress for females compared to males, such as the lower capacity to sweat and a higher body fat percentage.^28^ The behavioral factors may also affect the vulnerability to heat exposure. For example, females were reported to be more active in the household, which may put females at higher risk during heat waves.^28^^,^^29^ Our results also highlighted that the fractions of hospital admissions attributed to non-optimum temperatures were highest in children and adolescents. Compared to adults, children and adolescents exhibit a distinct susceptibility to heat exposure due to their diminished capacity for core body temperature regulation and their increased engagement in outdoor activities.^30^^,^^31^ Notably, our findings suggested that cold temperatures exert a more pronounced health burden in older adults (60–79 and 80+ years) as compared to other age groups. This may be related to the impaired thermoregulatory ability and a higher prevalence of underlying conditions among elderly individuals.^32^ These findings also demonstrate that cold-related health impacts still shouldn't be ignored and targeted policies should be developed for both genders and different age groups.

On the global scale, non-optimum temperatures could disproportionately affect the health burden in some regions.^3^ Similarly, substantial geographical disparities in hospital admissions attributable to non-optimum temperatures were observed in our study. A higher burden of hospital admissions was observed in Southern Thailand for both inpatients and outpatients. Various demographic and socioeconomic characteristics could contribute to the spatial heterogeneity, such as population density, education level, community infrastructures, and GDP per capita.^33^ In addition, differences in local climate conditions may also lead to differentiated health effects due to non-optimum temperatures.^34^ For example, our analysis showed that the effect estimates for extreme cold and hot temperatures in provinces with a wet climate were lower than those with a dry climate (supplementary Fig. S3). The assessment of vulnerable areas and populations are critical elements of adaptation strategies to climate change, which could provide comprehensive evidence to strengthen the resilience of the existing health system.^27^^,^^35^

We found evidence that a substantial burden of hospital admissions could be attributed to non-optimum temperatures for all diseases, with variations observed among different causes. As found by our results, for cardiovascular diseases and mental illness, outpatients were more likely to be attributed to hot temperatures while inpatients were more affected by cold temperatures. The effects of cold temperatures on the cardiovascular system are often due to potential complications in relation to changes in the autonomic nervous system, blood pressure, thermogenesis, inflammatory response, and oxidative stress.^18^^,^^36^^,^^37^ Similarly, exposure to extreme high temperatures could induce elevated heart rate, blood viscosity and plasma cholesterol levels, reduced cerebral perfusion, attenuated vasoconstrictor responsiveness, and alternation of fluid and electrolytic balance.^18^^,^^36^^,^^37^ Our results suggest that cold exposure is more likely to exacerbate the risk of hospitalization (inpatient) for cardiovascular diseases compared to heat exposure, which tends to cause mild cardiovascular symptoms (increased risk of outpatient visits). We also observed that the majority of outpatient admissions for endocrine, nutritional and metabolic diseases could be attributed to hot temperature. It was suggested that patients with diabetes tend to have impaired thermoregulatory capacity, which makes them more vulnerable to heat exposure.^38^ We identified that the attributable fraction of outpatient visits was the highest for musculoskeletal diseases. It was previously documented that heat exposure may lead to metabolic derangements and increase inflammation in the skeletal muscles.^39^ Besides, we found infectious and parasitic diseases have the largest attributable fraction for inpatient visits. Another study reported that non-optimum temperatures elevated the risk of hand, foot, and mouth disease in East China, which may be related to behavioral factors and the increase in the replication and survival of pathogens or vectors.^40^^,^^41^ However, the exact etiological pathways between non-optimum temperatures and most of the causes remain unclear. Our findings could provide novel insights regarding the health burden of potential causes associated with non-optimum temperatures. More studies are warranted to identify the impacts of ambient temperature on different diseases and unveil the potential mechanism in the future.

Our study has several strengths. First, this is the first national study assessing the morbidity impacts of non-optimum temperatures in Thailand. Our findings provide comprehensive evidence of the impacts on all-cause and cause-specific hospital admissions, which could mitigate the influence on healthcare utilization and inform future policies. Second, the population-weighted temperature was used in this study, which enhanced the precision of population exposure estimations.^42^^,^^43^ Third, our investigations also provided evidence of morbidity burden in different sex and age subgroups, which could help to identify the vulnerable populations.

Some limitations should be acknowledged. First, it should be cautious in interpreting our estimations because the current investigation was an ecological study, which was subject to individual-level confounders and unmeasured area-level variables. Therefore, the estimated risks and attributable fractions due to non-optimum temperatures should be interpreted cautiously. Second, our analysis used gridded temperature data rather than individual-level temperature data, which might lead to random measurement error. However, this error is more likely to be Berkson type error, which primarily reduces the study's power without attenuating risk estimates.^44^^,^^45^ Third, the diagnoses of all diseases were dependent on the ICD-10 codes, which may lead to coding errors and outcome misclassifications. Fourth, while societal adaptation measures like air conditioning access, healthcare system improvements, early warning systems, and socioeconomic well-being could potentially alter the temperature-hospitalization association, data limitations at the province level preclude directly assessing their impacts.^46^^,^^47^ Future research with access to such data can shed light on how these factors modify the temperature-hospitalization association and inform adaptation strategies. In addition, the current study covers 76% of the Thai population, which may lead to a selection bias. However, this is still the largest nationwide study in Thailand and could provide evidence for policy makers to develop relative strategies. Finally, the data used in this study only covered the period from 2013 to 2019. However, excluding data from 2020 to 2022 was necessary to prevent potential confounding effects induced by the coronavirus disease 2019 (COVID-19) pandemic.^48^ During this period, governments implemented extensive containment measures, including quarantines, lockdowns, travel restrictions, and resource reallocation, to curb viral spread and reduce mortality.^49^ These measures, both directly and indirectly, had a significant influence on temperature-related hospitalization rates.^48^ For example, stay-at-home orders would curtail outdoor physical activity, therefore decreasing exposure to non-optimum temperatures.^50^ The COVID-19 pandemic also caused a shortage of healthcare resources, leading to increased vulnerability of patients to non-optimum temperatures.^48^^,^^51^ Additionally, increased risk of mental illness during this period could have negatively impacted overall health outcomes.^52^

## Conclusion

The results indicate that low and high temperature has a significant impact on hospital admissions, especially among the females, children and adolescents (0–19 years). Our findings provide evidence for policymakers to develop adaptation strategies that can mitigate the adverse effects of climate change on public health in Thailand and other tropical countries. This study implicated that heat exposure has become a primary concern for public health in tropical countries and further highlighted the urgency to assess the vulnerability at the national level and address challenges associated with future extreme temperatures.

## Contributors

Conceptualization, methodology, validation: BW, WK, KT; Investigation: BW, HA, WK; Formal analysis, visualization: BW and WK; Writing original draft: BW, HA, WK; Writing-review and editing: BW, HA, WK, SS, NS, WS, SK; Critical comments provide: YG, KT; BT, RX, SL; Study supervision provide: YG, KT, BT, RX, SL; Project administration: YG, KT; All authors approved the paper for submission.

## Data sharing statement

The authors are not allowed to share the data used in this study.

## Editor note

The Lancet Group takes a neutral position with respect to territorial claims in published maps and institutional affiliations.

## Declaration of interests

The authors declare they have no competing interests.
