# Supplementary material for: Association of cause-specific hospital admissions with high and low temperatures in Thailand: a nationwide time series study
Source: Lancet Reg Health West Pac. 2024 Apr 4;46:101058. doi: 10.1016/j.lanwpc.2024.101058 (PMC11000193; doi:10.1016/j.lanwpc.2024.101058)
Supplement: Supplementary Figures and Tables [file mmc1.docx]

**Association of cause-specific hospital admissions with high and low temperatures in Thailand: a nationwide time series study**

**Supplementary material**

**Table of contents**

[Table S1. The relative risk (RR) of hospital admissions at the extreme cold temperature and extreme hot temperature compared with the MRT. 3](#_Toc159359682)

[Figure S1. The cause-specific exposure-response association between temperature and outpatient hospital admission in Thailand. 4](#_Toc159359683)

[Figure S2. The cause-specific exposure-response association between temperature and inpatient hospital admission in Thailand. 5](#_Toc159359684)

[Table S2. Sensitivity analyses by changing the maximum number of days for the lag period. 6](#_Toc159359685)

[Table S3. Sensitivity analyses by changing the df for the long-term trend and seasonality. 7](#_Toc159359686)

[Table S4. Sensitivity analyses by including diurnal temperature range (DTR) in the model. 8](#_Toc159359687)

[Table S5. Sensitivity analyses by using penalized spline function for relative humidity in the model. 9](#_Toc159359688)

[Figure S3. The exposure-response association between temperature and hospital admission in Thailand, stratified by climate (wet and dry).. 10](#_Toc159359689)

## Table S1. The relative risk (RR) of hospital admissions at the extreme cold temperature and extreme hot temperature compared with the MRT.

|  | Outpatient | | |  | Inpatient | | |
| --- | --- | --- | --- | --- | --- | --- | --- |
|  | MRT (˚C) | Extreme cold (95% CI) | Extreme hot (95% CI) |  | MRT (˚C) | Extreme cold (95% CI) | Extreme hot (95% CI) |
| **All** | 26.4 | 1.07 (1.03, 1.11) | 1.24 (1.19, 1.29) |  | 27.2 | 1.07 (1.03, 1.11) | 1.11 (1.07, 1.14) |
| **Sex** |  |  |  |  |  |  |  |
| Male | 26.4 | 1.07 (1.03, 1.11) | 1.21 (1.16, 1.27) |  | 27.4 | 1.07 (1.04, 1.11) | 1.10 (1.07, 1.14) |
| Female | 26.3 | 1.07 (1.03, 1.11) | 1.26 (1.20, 1.31) |  | 27.1 | 1.06 (1.02, 1.10) | 1.11 (1.08, 1.14) |
| **Age** |  |  |  |  |  |  |  |
| 0–19 years | 18.5 | 1.03 (1.02, 1.04) | 1.17 (1.12, 1.22) |  | 18.5 | 1.05 (1.04, 1.07) | 1.15 (1.10, 1.21) |
| 20–39 years | 26.5 | 1.08 (1.04, 1.11) | 1.19 (1.15, 1.24) |  | 27.1 | 1.09 (1.05, 1.13) | 1.08 (1.04, 1.13) |
| 40–59 years | 26.4 | 1.07 (1.03, 1.12) | 1.27 (1.21, 1.33) |  | 26.8 | 1.04 (1.01, 1.07) | 1.12 (1.08, 1.16) |
| 60–79 years | 26.3 | 1.10 (1.05, 1.15) | 1.30 (1.24, 1.37) |  | 27.3 | 1.10 (1.06, 1.15) | 1.11 (1.08, 1.15) |
| 80+ years | 26.1 | 1.11 (1.06, 1.16) | 1.28 (1.22, 1.35) |  | 27.1 | 1.14 (1.09, 1.19) | 1.10 (1.06, 1.15) |

MRT: minimum risk temperature; Extreme cold: 5th percentile of the temperature distribution; Extreme hot: 95th percentile of the temperature distribution





## Figure S1. The cause-specific exposure-response association between temperature and outpatient hospital admission in Thailand.





## Figure S2. The cause-specific exposure-response association between temperature and inpatient hospital admission in Thailand.

## Table S2. Sensitivity analyses by changing the maximum number of days for the lag period.

|  | Outpatient | | |  | Inpatient | | |
| --- | --- | --- | --- | --- | --- | --- | --- |
|  | MRT (˚C) | Extreme cold (95% CI) | Extreme hot (95% CI) |  | MRT (˚C) | Extreme cold (95% CI) | Extreme hot (95% CI) |
| Lag = 19 | 18.5 | 1.03 (1.01, 1.04) | 1.23 (1.17, 1.29) |  | 27.0 | 1.03 (1.00, 1.06) | 1.11 (1.08, 1.14) |
| Lag = 20 | 26.2 | 1.04 (1.00, 1.08) | 1.24 (1.19, 1.29) |  | 27.1 | 1.05 (1.02, 1.09) | 1.11 (1.08, 1.14) |
| Lag = 21 | 26.4 | 1.07 (1.03, 1.11) | 1.24 (1.19, 1.29) |  | 27.2 | 1.07 (1.03, 1.11) | 1.11 (1.07, 1.14) |
| Lag = 22 | 26.5 | 1.08 (1.03, 1.12) | 1.23 (1.17, 1.28) |  | 27.3 | 1.07 (1.04, 1.11) | 1.10 (1.06, 1.13) |
| Lag = 23 | 26.5 | 1.07 (1.03, 1.12) | 1.24 (1.18, 1.30) |  | 27.4 | 1.08 (1.04, 1.12) | 1.10 (1.06, 1.14) |

MRT: minimum risk temperature; Extreme cold: 5th percentile of the temperature distribution; Extreme hot: 95th percentile of the temperature distribution.

## Table S3. Sensitivity analyses by changing the df for the long-term trend and seasonality.

|  | Outpatient | | |  | Inpatient | | |
| --- | --- | --- | --- | --- | --- | --- | --- |
|  | MRT (˚C) | Extreme cold (95% CI) | Extreme hot (95% CI) |  | MRT (˚C) | Extreme cold (95% CI) | Extreme hot (95% CI) |
| Df = 6 | 18.5 | 1.04 (1.03, 1.06) | 1.18 (1.13, 1.24) |  | 26.9 | 1.10 (1.07, 1.14) | 1.09 (1.06, 1.12) |
| Df = 7 | 26.2 | 1.06 (1.02, 1.09) | 1.14 (1.09, 1.18) |  | 27.1 | 1.11 (1.08, 1.14) | 1.09 (1.06, 1.12) |
| Df = 8 | 26.4 | 1.07 (1.03, 1.11) | 1.24 (1.19, 1.29) |  | 27.2 | 1.07 (1.03, 1.11) | 1.11 (1.07, 1.14) |
| Df = 9 | 25.4 | 1.05 (1.01, 1.09) | 1.23 (1.17, 1.28) |  | 25.6 | 1.03 (1.00, 1.06) | 1.10 (1.06, 1.14) |
| Df = 10 | 25.4 | 1.06 (1.02, 1.11) | 1.26 (1.21, 1.32) |  | 25.5 | 1.04 (1.01, 1.07) | 1.10 (1.07, 1.14) |

MRT: minimum risk temperature; Extreme cold: 5th percentile of the temperature distribution; Extreme hot temperature: 95th percentile of the temperature distribution.

## Table S4. Sensitivity analyses by including diurnal temperature range (DTR) in the model.

|  | Outpatient | | |  | Inpatient | | |
| --- | --- | --- | --- | --- | --- | --- | --- |
|  | MRT (˚C) | Extreme cold (95% CI) | Extreme hot (95% CI) |  | MRT (˚C) | Extreme cold (95% CI) | Extreme hot (95% CI) |
| Main model | 26.4 | 1.07 (1.03, 1.11) | 1.24 (1.19, 1.29) |  | 27.2 | 1.07 (1.03, 1.11) | 1.11 (1.07, 1.14) |
| Main model + DTR | 26.4 | 1.07 (1.03, 1.12) | 1.24 (1.19, 1.30) |  | 27.2 | 1.07 (1.04, 1.11) | 1.11 (1.07, 1.14) |

MRT: minimum risk temperature; Extreme cold: 5th percentile of the temperature distribution; Extreme hot: 95th percentile of the temperature distribution; DTR: diurnal temperature range.

## Table S5. Sensitivity analyses by using penalized spline function for relative humidity in the model.

|  | Outpatient | | |  | Inpatient | | |
| --- | --- | --- | --- | --- | --- | --- | --- |
|  | MRT (˚C) | Extreme cold (95% CI) | Extreme hot (95% CI) |  | MRT (˚C) | Extreme cold (95% CI) | Extreme hot (95% CI) |
| Main model | 26.4 | 1.07 (1.03, 1.11) | 1.24 (1.19, 1.29) |  | 27.2 | 1.07 (1.03, 1.11) | 1.11 (1.07, 1.14) |
| Penalized spline | 26.4 | 1.06 (1.02, 1.10) | 1.24 (1.19, 1.30) |  | 27.2 | 1.06 (1.03, 1.10) | 1.11 (1.07, 1.14) |

MRT: minimum risk temperature; Extreme cold: 5th percentile of the temperature distribution; Extreme hot: 95th percentile of the temperature distribution; DTR: diurnal temperature range.

**
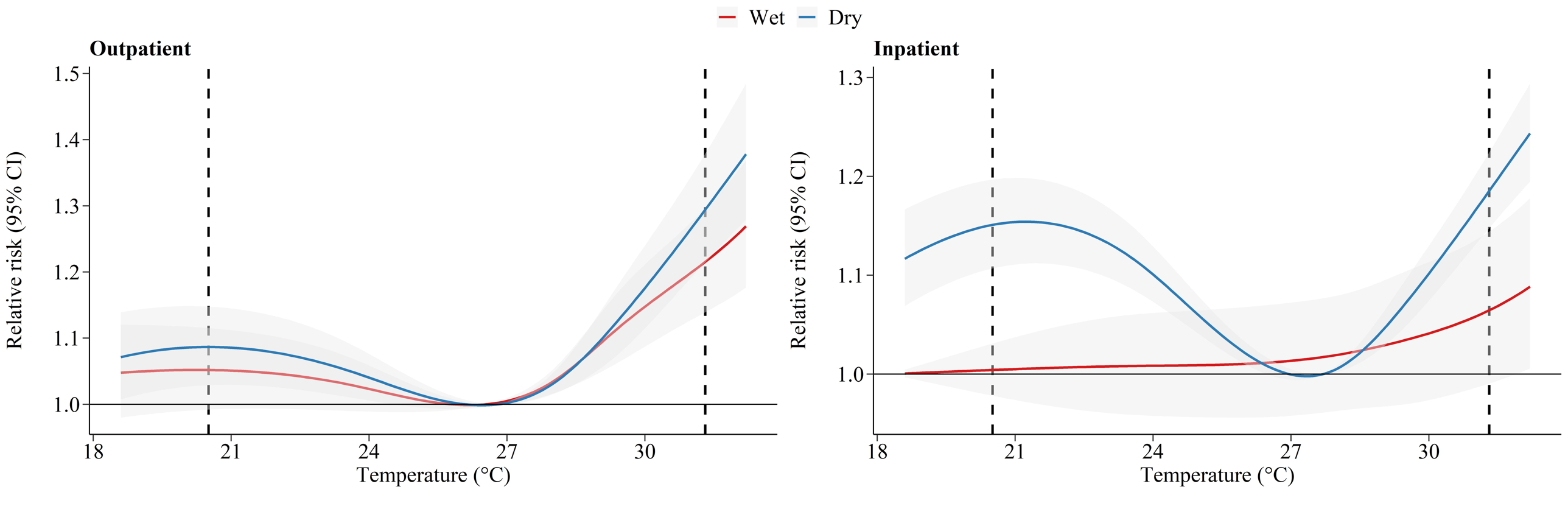
**

## Figure S3. The exposure-response association between temperature and hospital admission in Thailand, stratified by climate (wet and dry). The provinces with a wet climate were defined as the relative humidity (RH) above the median (50%) of province-specific average, while those with a dry climate were defined as the RH below the median (50%) of province-specific average.
